# Tactile shape discrimination for moving stimuli

**DOI:** 10.1038/s41598-024-58509-6

**Published:** 2024-04-15

**Authors:** Nicolas Pélegrin, Mahiko Konishi, Jean-Christophe Sarrazin

**Affiliations:** 1 ONERA, Information Processing and Systems Department, Cognitive Engineering and Applied Neurosciences Research Unit, Salon-de-Provence, 13661 France; 2 Jellysmack, Paris, 75008 France

**Keywords:** Somatosensory system, Perception, Sensory processing

## Abstract

In this study, we explored spatial-temporal dependencies and their impact on the tactile perception of moving objects. Building on previous research linking visual perception and human movement, we examined if an imputed motion mechanism operates within the tactile modality. We focused on how biological coherence between space and time, characteristic of human movement, influences tactile perception. An experiment was designed wherein participants were stimulated on their right palm with tactile patterns, either ambiguous (incongruent conditions) or non-ambiguous (congruent conditions) relative to a biological motion law (two-thirds power law) and asked to report perceived shape and associated confidence. Our findings reveal that introducing ambiguous tactile patterns (1) significantly diminishes tactile discrimination performance, implying motor features of shape recognition in vision are also observed in the tactile modality, and (2) undermines participants’ response confidence, uncovering the accessibility degree of information determining the tactile percept’s conscious representation. Analysis based on the Hierarchical Drift Diffusion Model unveiled the sensitivity of the evidence accumulation process to the stimulus’s informational ambiguity and provides insight into tactile perception as predictive dynamics for reducing uncertainty. These discoveries deepen our understanding of tactile perception mechanisms and underscore the criticality of predictions in sensory information processing.

## Introduction

Misestimations in tactile perception have been highlighted by various studies, illustrating how cognitive factors can influence our interpretation of tactile stimuli. A recent study conducted by Perquin et al.^1^ studied individuals’ ability to perceive complex tactile stimuli on the palm of the hand. They generated stimuli involving movement in three different directional axes: horizontal, vertical, and oblique on the palm’s surface. They demonstrated that the participants’ perceived tactile movement direction exhibited a directional bias, such that movement along the cardinal horizontal and vertical axes of the hand was perceived much more easily and confidently than movement aligned with an oblique axis. This finding extends the classic oblique effect^2^ observed in visual perception to the realm of tactile perception and reveals the amodal nature, independent of sensory modality, of this cognitive bias, dependent on information processing involved in motion perception.

The phenomena of interdependence between spatial and temporal dimensions that characterize perception are a clear example of this. Their effects on the misestimation of movement have been demonstrated repeatedly in well-known phenomena in the literature, such as the cutaneous rabbit^3–5^, as well as Tau^6^ and Kappa effects^7^. The cutaneous rabbit derives its name from the idea that tactile stimuli applied to the skin can give the impression of a rabbit jumping from one body region to another. This perceptual illusion is generated by applying two successive tactile stimulations to the skin at very short time intervals, on the order of 50–80 milliseconds. If the stimulations are applied regularly, the nervous system tends to integrate them into a coherent sensation of movement, creating the impression that the stimulus is moving across the skin between the two positions. The Tau effect, on the other hand, is another phenomenon of interdependence between space and time in tactile perception. Previous work by Lechelt and Bochert^8^ in 1977 demonstrated that the perception error of spatial properties of perceived movement depends on the temporal context of stimulation. They generated two stimuli on each forearm separated by a spatial interval of 10 cm. The forearms were stimulated successively with a delay of 1500 ms, and the duration of the temporal interval varied from one forearm to the other. Participants were asked to judge whether the second spatial interval was larger or smaller than the first, and their judgment could vary by twice as much depending on the t1/t2 ratio. Ultimately, these two phenomena of interdependence between space and time can affect our tactile perception in various ways. One possible interpretation of these illusions would be that perception is not a simple reproduction of reality but rather an interpretation constructed by our nervous system from the available sensory information.

Studies have explored the cognitive mechanisms behind this perceptual bias. The commonly accepted integration of spatial and temporal dimensions suggests that participants attribute movement to the discontinuous displacement of the target. Specifically, according to Collyer^9^, considering two spatial intervals (s1 and s2) and two temporal intervals (t1 and t2) traversed at speeds v1 and v2, participants equalize the ratios s1/s2 and t1/t2. This implies that s1/t1 = s2/t2 (or v1 = v2). Except for the notable constant velocity model provided by Jones and Huang^10^, for which it is not very clear how to apply it to sequences of more than two space-time intervals, very few models have been formalized to account for these spatiotemporal dependencies. In this context, Sarrazin, Tonnelier and Alexandre^11^ developed a model that reformulates the constant velocity hypothesis in the context of more complex sequences (more than three stimuli) than those traditionally studied. They showed that the reformulation of the constant velocity hypothesis can reproduce the behavior observed in the Tau experiment. However, by introducing a new hypothesis based on the minimization of neighboring velocity variations, inspired by works from both the perception and motor control domains (trajectory formation in particular), they improved the performance of the motion attribution model^11^.

Building upon previously discussed principles of minimizing velocity variations, the research by Faugloire et al.^12^, focusing on the recognition of two-dimensional vibrotactile patterns on the abdomen, highlighted the significance of spatial and temporal separation of elements for optimizing recognition. This study not only revealed that increased isolation between elements in space and time enhances recognition accuracy but also emphasized the substantial influence of the presentation speed of patterns on tactile recognition capabilities. This underscores the critical role of speed in the efficiency of tactile information transmission and its interaction with spatial and temporal factors in tactile perception. Furthermore, the work of Faugloire et al.^12^ complements this perspective by illuminating the importance of speed in tactile perception, thereby establishing a fundamental commonality with the motion attribution model. Speed, as a key factor in optimizing tactile recognition, resonates with the principle of minimizing velocity variations for a more uniform perception of motion. This convergence suggests that the imputed motion^10^ we perceive might be influenced by similar perceptual mechanisms, where smoothness and continuity of motion are favored, in both visual and tactile perception. Thus, by integrating the findings of Faugloire et al.^12^, we bolster the notion that the principles governing perception and motor control extend beyond the visual domain to also encompass the tactile domain, enriching our understanding of imputed motion within a multisensory framework.

In the field of motor control, it has been presumed that a major challenge in our understanding of motor coordination is the study of producing as smooth a movement as possible by minimizing changes in the effector’s accelerations (e.g., the hand). The minimum-jerk model^13^ predicts the smoothest possible trajectory for a class of human movements and provides a qualitative measure of its kinematics. The acceleration minimization model^11^ follows this argument in that it homogenizes the apparent speed of perception through the minimization of neighboring velocity variations. This similarity suggests that general principles govern both perception and motor skills, as defined by Viviani and Stucchi^14^ in the context of motor theories of perception. These theories suggest that our perception of movement is influenced by motor patterns, cognitive models that represent the actions and movements we perform or observe. By using these motion laws, our nervous system can predict the evolution of a moving object and anticipate its future position.

The internal relationships between action and perception have also been highlighted through a biological law of movement: the two-thirds power law^15^. Early research^16–19^ on handwriting and drawing movements revealed a relationship between the execution speed of the final effector and the trajectory that is generated. The results of these studies found that the angular velocity $V(t)$ of the movement is proportional to the curvature $R(t)$ of the final effector’s trajectory. These studies mathematically formulated this relationship of motor trajectories by establishing that the movement’s speed, denoted as $V(t)$, can be represented by the equation $V(t) = K \times \left[ \frac{R(t)}{1 + \alpha R(t)} \right] ^\beta$. In this equation, $\beta$ corresponds to the exponent of the power law, the factor $K$ is a constant representing the speed gain and the parameter $\alpha$ ranges between 0 and .1, depending on the average velocity. This law expresses a relationship between the geometric and temporal aspects of human movement in the motor domain. However, this law has also been observed in the field of visual perception of moving objects^14,20,21^. Viviani and Stucchi^14^ showed that, with a cursor describing a circular trajectory and a constant tangential displacement speed, subjects perceived a circular shape on their screen. Similarly, when a cursor describing an elliptical trajectory was presented with a tangential displacement speed that varied according to the radius of curvature, subjects perceived an elliptical shape on their screen. However, when the stimulus was characterized by informational incongruence, meaning that the tangential displacement speed of the cursor no longer followed the principle of the two-thirds power law, subjects were more likely to make misperceive the stimulus. Thus, if a cursor described a circular trajectory with a displacement speed corresponding to that of the ellipse, subjects were more likely to perceive an elliptical shape rather than a circular one, and vice versa.

Upon closer examination of the impact of informational ambiguity, it becomes evident that it not only influences our tactile perception but also shapes our metacognition, that is, our ability to assess and judge our own perception. Incongruent tactile stimuli, characterized by greater informational ambiguity, can pose additional challenges to our metacognitive system. Faced with contradictory or ambiguous information, we may wonder to what extent we can be confident in our perceptions. The study of metacognition in the context of sensory perception is a research domain where researchers are interested in how we evaluate our own perception and the accuracy of these evaluations^22,23^. Previous work has shown that our metacognition can be influenced by various factors, such as the clarity of sensory stimuli, past experience, and even cognitive biases^24,25^. In our study, we will examine how informational ambiguity affects not only tactile perception itself but also the confidence we place in our perceptual judgments and associated metacognitive levels.

This work focuses on how spatiotemporal dependencies can modify the tactile perception of a moving object. Building on the work of Viviani and Stucchi^14^, we explore whether the motion attribution mechanism also operates in the tactile modality and, if so, how this mechanism modulates the processing of informational incongruence in stimulations. To address this question, we designed an experiment where we stimulated the right palm’s surface of participants with four different tactile pattern configurations, including two congruent configurations and two incongruent configurations. Congruent stimuli are characterized by a circular shape with a constant displacement speed and an elliptical shape with a displacement speed varying in accordance with the ’two-thirds power law’. Incongruent stimuli, on the other hand, reverse the temporal characteristics of spatial trajectories. In this experiment, we asked participants to distinguish whether they perceived a circle or an ellipse among the tactile patterns and then evaluate their confidence level in their response. The underlying hypothesis of our study is that incongruent stimuli, characterized by greater informational ambiguity, may lead to reduced precision in the discrimination of perceived tactile patterns. This can also affect participants’ confidence in their perceptual responses, thus influencing their level of metacognitive sensitivity. We assume that when sensory information becomes more complex or contradictory, metacognition can play a crucial role in influencing how we interpret these tactile sensations and evaluate our confidence in our own perception. By studying these complex relationships between informational ambiguity, tactile perception, and metacognition, we aim to shed light on the underlying mechanisms of human perception and improve our understanding of how we process complex sensory information. This approach will also help explore how metacognitive processes interact with tactile perception, thus providing fresh insights into motion perception and its links with our sensory awareness.

## Results

### Performance

In this section, we will present the results of our participants’ performance in the analysis of shape, independent of the speed of movement, as well as in the analysis of speed itself. Subsequently, we will address the effect of congruence between stimuli and, finally, the combined effect of shape and speed.

To analyze the effect of shape independently of the speed of movement, we compared discrimination performance between circle and ellipse-shaped stimuli. The results of this analysis did not reveal a significant difference, with a Wilcoxon test yielding W = 257.5 and p = 0.123. The mean discrimination accuracy was 0.585 for circle-shaped stimuli and 0.547 for ellipse-shaped stimuli. We also examined the effect of the speed of tactile stimulus movement by comparing performance between constant and variable speed stimuli. The results of this analysis did not show a significant difference, with a Wilcoxon test yielding W = 256.5 and p = 0.13. The mean discrimination accuracy was 0.585 for constant speed stimuli and 0.565 for variable speed stimuli.

**Figure 1:**
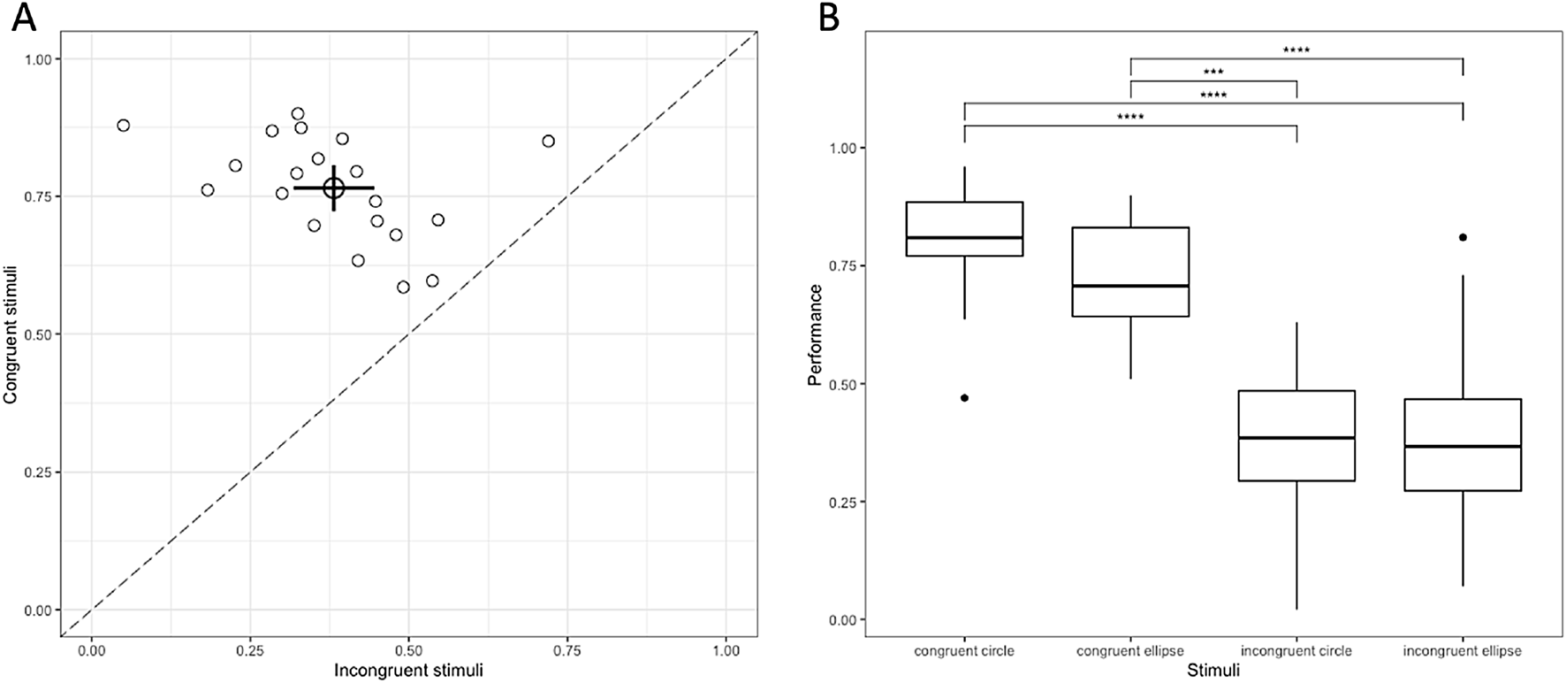
Effect of temporal pattern on tactile perception. (**A**) Scatter plot of performance between congruent and incongruent stimuli. (**B**) Multiple comparison test using the paired Wilcoxon signed-rank test on tactile stimuli.

Next, we assessed the effect of congruence by comparing performance between congruent and incongruent stimuli. The results of the experiment revealed a significant effect of congruence on tactile shape perception. The discrimination rate was 77% for congruent cases and 38% for incongruent cases (Fig. 1A). A Wilcoxon test demonstrated a statistically significant effect between congruent and incongruent stimuli (W = 7, p < 0.001), with a large effect size detected (r = 0.825).

Finally, we explored the combined effect of shape and speed by individually studying each stimulus in each condition. For each stimulus, we analyzed discrimination performance while considering the speed of movement (Fig. 1B). The results showed a significant effect of shape and speed factors on subject performance (X2(3) = 42.2, p < 0.001). The detected effect size was high (W = 0.707). Moreover, we conducted a multiple comparison test using the paired Wilcoxon signed-rank test to examine differences between each stimulus. The results revealed that the two congruent stimuli were not statistically different. However, they were significantly different from the two other incongruent stimuli.

We also considered within-subject fluctuations in performance by checking if they were caused by fluctuations in hand temperature. The results of this analysis showed that performance fluctuations were not significantly related to hand temperature variations, eliminating a potential bias factor in our observations (p = 0.555).

### Confidence—metacognitive efficiency

Participants rated their confidence in their responses on a 4-point scale. A significant difference in confidence was observed between congruent and incongruent stimuli according to the Wilcoxon test (p < 0.001). In the presence of a congruent stimulus, confidence in the response was higher than that observed for an incongruent stimulus.

To assess participants’ metacognitive efficiency in each condition (congruent and incongruent), we calculated the $M_{ratio}$ for each participant. The results showed an $M_{ratio}$ of 0.72 for congruent stimuli, indicating that participants use approximately 72% of the available information in the percept to make their response. In contrast, for incongruent stimuli, the $M_{ratio}$ was 0.55, revealing that participants use only 55% of the available information to respond in this case. This suggests that responses provided by participants in the incongruent case are accompanied by a higher level of uncertainty than in the congruent case. Despite this difference in metacognitive efficiency between congruent and incongruent conditions, the Wilcoxon test did not reveal a significant difference between the two conditions (p = 0.277). Additionally, we noted that the incongruent condition appears to produce a wider variety of $M_{ratio}$ values, indicating that participants are more heterogeneous in their use of available information to make decisions in this condition. To bolster our results, we also performed a Bayes Factor measure on a paired t-test between the two conditions ($BF_{10}$ = 0.44). The result indicates that we do not have sufficient evidence to affirm that the congruent and incongruent conditions are truly different in terms of metacognitive efficiency.

### HDDM modeling of response times

To better understand the underlying mechanisms of participants’ response times in congruent and incongruent conditions, we conducted HDDM modeling. In this modeling, we used the absolute value of drift rate for all analyses in the different conditions.

**Figure 2:**
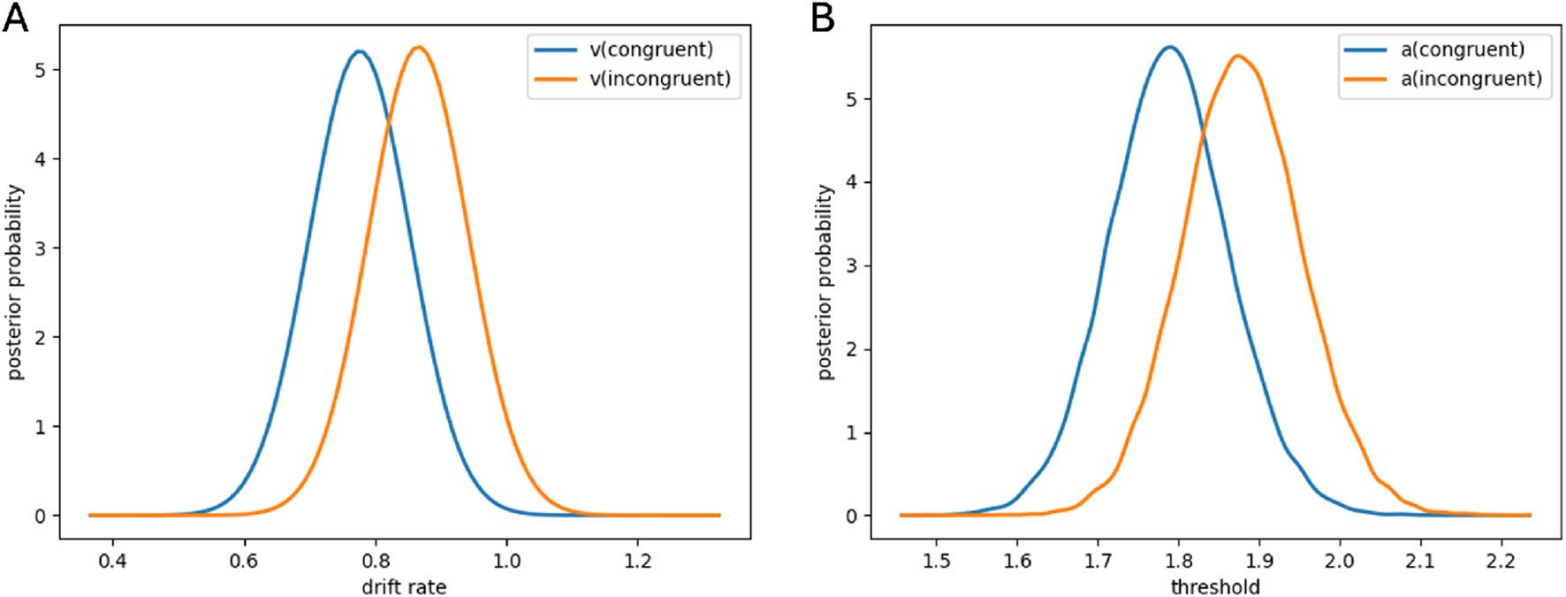
Comparison of mean posterior densities of drift rates (**A**) and decision thresholds (**B**) between congruent and incongruent conditions.

We individually adapted the HDDM model for each participant (Fig. 2) and used a paired t-test to examine differences in drift rates and decision thresholds between congruent and incongruent conditions, adopting a within-subject approach. Contrary to our initial observations, this analysis revealed no significant difference in drift rates between the two conditions (p = 0.2372). Nevertheless, we identified a significant effect on the decision threshold (p < 0.0001), where the threshold was notably higher in the incongruent condition.

**Figure 3:**
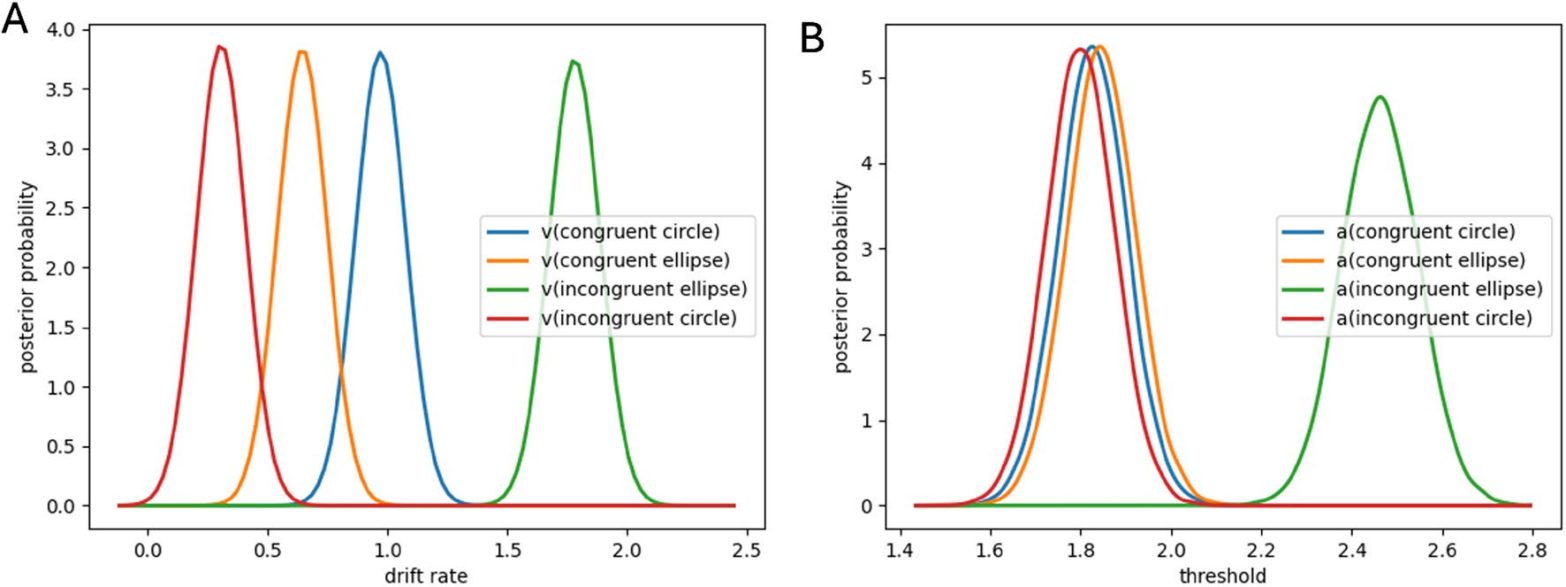
Analysis of drift rate (**A**) and decision threshold (**B**) based on congruence and stimulus shape.

We furthered our analysis by adapting the model to the four experimental conditions (Fig. 3): congruence/incongruence crossed with the shape of the stimuli (circular or elliptical). This approach allowed us to more precisely distinguish the discrimination processes at play according to the types of stimuli. Our statistical analyses, based on a repeated measures ANOVA, revealed significant differences for both drift rate and decision threshold (F(3,57) = 64.95 and p < 0.0001 for drift rate; F(3,57) = 111.29 and p < 0.0001 for decision threshold), indicating substantial effects of our experimental conditions on these parameters. Regarding drift rate, post hoc analyses adjusted by Bonferroni showed no significant difference between congruent stimuli (p = 0.064). However, the incongruent elliptical stimulus stood out distinctly from all others (p < 0.0001 in all cases), highlighting a peculiarity in the processing of this type of stimulus. No significant difference was observed between the congruent elliptical stimulus and the incongruent circular stimulus (p = 0.286). As for the decision threshold, post hoc results, also adjusted by Bonferroni, revealed that thresholds were significantly lower in both congruent conditions as well as for the incongruent circular stimulus, showing no significant difference among them (p = 1). In contrast, the threshold for the incongruent elliptical stimulus was significantly higher, compared to each of the other conditions (p < 0.0001 in all cases).

## Discussion

In this study, we investigated spatiotemporal dependencies and their influence on the tactile perception of moving objects. Our work built on prior research that demonstrated how relationships between execution speed and effector trajectory, which characterize human movements, also condition the visual perception of movements^14,15^. Specifically, we sought to determine if the motor features of shape recognition, mainly studied in the visual modality, are also active in the tactile modality. Our research question aimed to understand how spatiotemporal dependencies alter the tactile perception of moving objects, especially when tactile stimulations have increased informational ambiguity. To address this, we designed an experiment where participants’ palms were stimulated with congruent and incongruent tactile patterns. Participants were required to report the perceived shape generated by the tactile stimulation trajectory and their confidence level in their response. We hypothesized that incongruent tactile stimuli, characterized by informational ambiguity, would lead to a reduced performance in discriminating perceived tactile patterns and would influence participants’ confidence in their perceptual responses. This study aimed to shed light on the underlying mechanisms of tactile motion perception and how we interpret complex sensory information.

Firstly, let’s examine the results showing the presence of motion illusion in the tactile domain, thereby confirming a connection between features relevant to trajectory generation and the recognition of patterns, whether in the visual or tactile domain. These findings, combined with prior research such as the work published by Sarrazin et al.^11^ in 2005 and Faugloire et al.^12^ in 2022, suggest the possibility that a mechanism related to motion attribution may be implicated in these perceptual distortions. When congruent tactile stimuli were presented, characterized by biological coherence (the relationship between space and time dimensions determined by the two-thirds power law), participants showed high accuracy in discriminating tactile patterns. However, this accuracy significantly decreased when incongruent tactile stimuli, characterized by biological incoherence (no relationship between space and time dimensions), were introduced, creating informational ambiguities between space and time. This distortion, previously documented^4,26^, provides empirical support for the hypothesis that motor features of shape recognition in vision are characterized in the tactile modality as proposed by Viviani and Stucchi^14^, who posited that motor schemas may contribute to the formation of our perception, thereby suggesting a motor theory of perception. While our results support the idea of a connection between motor features and perception, it is important to consider critiques such as those presented by Bingham and Wickelgren^27^, who challenge the universality of motor theories of perception by highlighting the complexity and diversity of perceptual mechanisms beyond mere motor frameworks. In other words, our nervous system appears to interpret ambiguous sensory information based on expectations and predictions^28,29^. In this context, it’s plausible that motion attribution mechanisms also play an active role in the tactile domain, making predictions about tactile stimuli’s trajectory and nature. These results emphasize the role of an amodal mechanism in perception and highlight the significance of prediction mechanisms in processing sensory reafferences. They suggest that the brain’s processing of tactile reafferences is not a passive process but is actively engaged in predicting these sensory reafferences^30^. These predictions are based not only on immediate sensory characteristics but also on past experiences, expectations, and intentions^31^. This notion of sensory prediction has significant implications for understanding motion perception more broadly. It raises questions about how we interpret complex sensory information and how our brain manages to create a coherent perception, considering both raw sensory data and knowledge about the phenomenon generating it.

Having established that biological coherence influences tactile percept performance, it’s essential to delve deeper into this perception. Perception isn’t just about accuracy or error; it also has a subjective dimension that influences how we interpret and react to stimuli. This dimension was our further exploration focus. Although our primary aim was to determine how spatiotemporal dependencies alter tactile perception of moving objects, we recognized that merely identifying shape or pattern would provide a limited view of the complete sensory experience. Confidence in perception is a crucial marker of subjective certainty and can offer insight into how individuals interpret and evaluate sensations. Confidence judgments about our responses are prototypical examples of metacognition. Metacognition is vital both for self-regulating cognition and for cooperation between agents in uncertain situations. It belongs to the executive functions that lend human cognition its flexible and adaptive character. With this perspective, we introduced confidence measurement as a window into the underlying mechanisms of subjective tactile perception experience. By assessing confidence, we can access a more nuanced perception dimension, giving us a more comprehensive view of the metacognitive processes shaping tactile experience. Based on this, we furthered our investigations by calculating the $M_{ratio}$. The $M_{ratio}$, as defined by Fleming and Lau^22^ and Maniscalco and Lau^32^, quantifies an observer’s ability to discriminate between correct responses and errors concerning their knowledge about the stimulus. In other words, it measures how aware an individual is of their cognitive abilities. While our statistical analyses didn’t reveal significant differences between congruent and incongruent conditions, an interesting trend emerged for incongruent stimuli (the average $M_{ratio}$ across participants was 0.72 for congruent stimuli and 0.55 for incongruent stimuli). Indeed, participants showed greater uncertainty when confronted with incongruous tactile stimuli. This observation suggests that the informational ambiguity introduced by incongruent stimuli could affect participants’ confidence in their responses. The trends observed in our study may indicate that more subtle differences are at play. Further research in this area would therefore be useful to better understand how informational ambiguity influences metacognition in the context of tactile motion perception.

A deeper analysis of the results, using the HDDM model^33^, provided further insights. The Hierarchical Drift Diffusion Model (HDDM) is an approach that allows us to better understand the underlying mechanisms of decision-making, especially in uncertain situations. The core of this approach rests on the idea that decision-making is not an instantaneous phenomenon, but rather a dynamic process of evidence accumulation. The distinctive advantage of the HDDM is its ability to model this mechanism based on information theory. Perception is seen here as a constant quest to reduce uncertainty. Information is accumulated until uncertainty is reduced to a level deemed acceptable for a response. This reduction in uncertainty is especially crucial as our perceptions are often time-constrained. This model gives us a precise representation of perception as a dynamic mechanism mainly aimed at accumulating information and reducing uncertainty. This modeling of perception is of crucial importance for our study as it sheds light on why the timing of a response can vary depending on stimuli and contexts. In our case, within-subject analyses did not reveal any significant difference in drift rates between congruent and incongruent conditions. This lack of difference applies to all stimuli, with the notable exception of the incongruent elliptical stimulus, which distinctly stands out from the others. However, it is noteworthy that in the incongruent condition, there is a slight elevation observed. A plausible explanation for this apparent contradiction lies in the decision threshold. This observation might indicate that the discrepancy between curvature and speed is used as information to judge the shape. In other words, participants might adopt a strategy whereby they decide immediately if no discrepancy is detected for a certain period of time, but if a discrepancy is detected, they monitor the situation for a while before deciding. This peculiarity highlights a specificity in the processing of this type of stimulus, suggesting a complexity in the tactile perception process. Otherwise, the HDDM showed that the decision threshold was higher in the incongruent condition. This suggests that in the congruent condition, the task is perceived as easier, leading to a significantly lower decision threshold. As a result, participants needed to accumulate less evidence to make their response, resulting in better performance and shorter response times. This observation aligns with the findings from our within-subject analyses on the decision threshold, where we observe a similar trend. These observations highlight a complex dynamic of the perception process in the tactile domain. While we do not observe significant divergence in the drift rate of stimuli, it is the decision threshold that reflects a greater confidence of participants in their responses in the presence of biologically plausible stimuli.

Ultimately, our results suggest that tactile perception of movement arises from a complex dynamic between a top-down mechanism, predictive control of action feedback, and a bottom-up mechanism for integrating sensory reafference similar to the concept of predictive coding^34–36^. This theory proposes a model of interaction between our intentions, our expectations, and the processing of sensory reafference to form a perception of the world we interact with. Predictive coding, a well-established theoretical framework in literature^37–40^, tells us that the central nervous system (CNS) generates predictions about the sensory consequences of stimulations at various integration levels^41^. Our intentions and expectations play a crucial role in this process. As demonstrated by Sarrazin, Cleeremans and Haggard^31^, if we expect to perceive movement in a given direction, our sensory predictions are adjusted accordingly. But this mechanism goes far beyond simple prediction based on expected stimuli. At the heart of this mechanism is a constant minimization of prediction error at every step of information processing. When the CNS perceives a stimulus, it tries to anticipate this information based on pre-existing internal models. If the prediction is correct, the error is minimal. But if it differs, a prediction error is detected. This error detection is then exploited to refine and improve our internal models, in an iterative process. Centrally, this dynamic is based on the Free Energy Principle theory^36^. It states that the CNS seeks to minimize prediction error. Thus, as information rises in the hierarchy of neural processing, the error tends to decrease. At the peak of this cascade, despite uncertainties and prediction errors that pre-exist at lower levels, a stable metacognitive representation emerges. In the case of our tactile stimulus, the CNS undergoes this cascade of predictive encodings of sensory information. If the stimulus aligns with the participant’s predictions, the prediction error is low. However, when a stimulus presents informational ambiguity, a greater prediction error can be suspected as it is not in line with the internal models from which predictions are made, in our case representations of human biological movement. These ambiguous situations increase uncertainty, as suggested by our data on the $M_{ratio}$. By integrating this concept, we see a relationship between sensory ambiguity and metacognitive performance. The more ambiguous a stimulus is, the higher the prediction error, thereby reducing the accessibility of information to the field of consciousness. Based on the works of Michel^24^, we discern a link between uncertainty (represented by the $M_{ratio}$), the amount of information used to make a response, and the ability of this information to penetrate the field of consciousness.

Drawing conclusions from our research, our study sheds light on the complex mechanisms of tactile movement perception, highlighting the interaction between perceptive performance and metacognitive confidence. Predictive coding is presented as essential in interpreting tactile stimuli, especially when facing informational ambiguity. By merging our findings with current literature, we deepen our understanding of the subjective experience generated by tactile movement, highlighting the importance of predictive mechanisms. These results, although preliminary, suggest potential applications in technological fields of mobility assistance and lay the foundation for future studies on perception and its temporality in the tactile context.

## Methods

### Participants

Twenty participants (10 females, 10 males), aged between 22 and 33 years, with a mean age of 27.2 years, were recruited from the laboratory members to participate in the experiment. All participants were right-handed and reported no tactile deficits in their dominant hand. Prior to participating in the experiment, participants provided informed consent. This study was conducted in accordance with the principles outlined in the Declaration of Helsinki and received approval from the Ethics Committee of Université Paris Cité (IRB No. 00012023-101). The experiment had a total duration of approximately one hour and forty-five-minute, which included instructions, breaks, and familiarization.

### Materials

**Figure 4:**
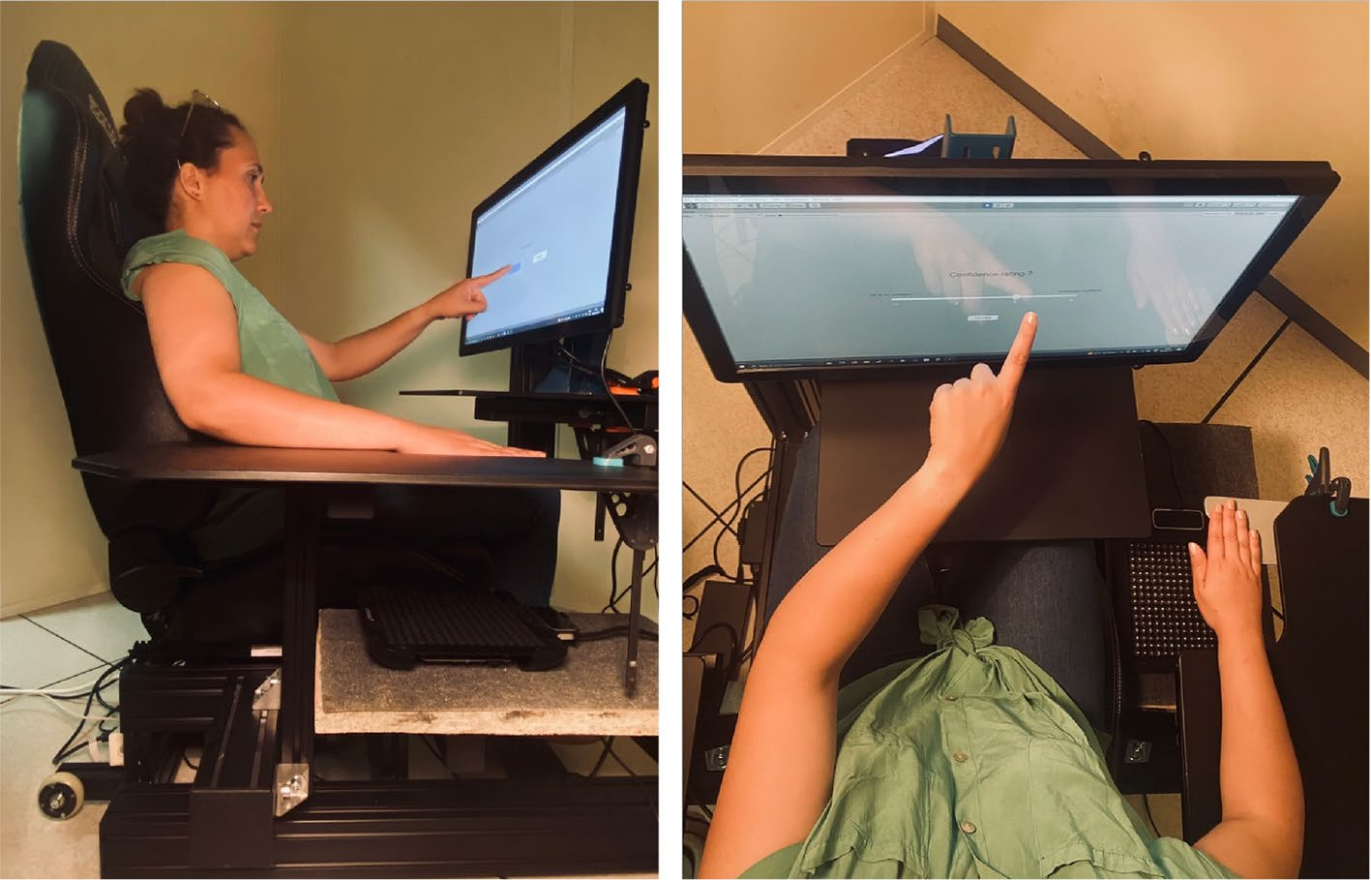
Overview of the experimental setup and participant positioning. Stimuli were delivered using an array of ultrasound actuators positioned 20 cm below the participant’s right hand.

The experiment was developed using Unity software and ran on a ZOTAC VR GO 2.0 computer. Tactile stimuli were generated using an UltraHaptics UHEV1 device (UltraLeap, Bristol, UK ; see Fig. 4). This device^42^ consists of a 16 $\times$ 16 array of transducers capable of producing airborne pressure points at ultrasound frequencies up to 40 kHz. Experiment management and data collection were facilitated by the open-source package “Unity Experiment Framework” (UXF, Brookes, 2020), while visual instructions and elements of the experiment were presented on an Elo Touch Solutions LCD Touchscreen Monitor (ET2294L, resolution: 1920 $\times$ 1080). Participant responses executed on the touchscreen monitor were recorded directly by UXF.

The palm skin temperature of participants’ right hands was measured for each participant before the start of each trial block (see section “Procedure”) using a FeverScan infrared medical thermometer (AX-IR22). This measurement was taken to ensure that variations in hand temperature remained minimal and did not influence the results of the tactile discrimination task. An audio headset was also used to mask the noise from the tactile stimulation device and prevent auditory disturbances during the experiment.

### Design

Each participant took part in a tactile discrimination task aimed at assessing their ability to distinguish different tactile patterns presented on the palm of their right hand. This task was performed under two different experimental conditions, allowing manipulation of both the shape and speed of tactile stimuli.

Regarding the shape of the stimulus, two tactile configurations were presented: a stimulus moving along a circular trajectory and another along an elliptical trajectory. The circular stimulus had a diameter of 4 cm, while the elliptical stimulus had a major axis of 4 cm and a minor axis of 2.8 cm.

Concerning the speed of stimulus movement, two conditions were employed: a constant speed and a variable speed following the two-thirds power law^15^. The two-thirds power law is an empirical relationship describing how the tangential velocity of a point moving in a two-dimensional plane varies with its radius of curvature along its trajectory. Mathematically, the law is expressed by the equation: $V(t) = K \times \left[ \frac{R(t)}{1 + \alpha R(t)} \right] ^\beta$ where $V(t)$ represents tangential velocity, $R(t)$ is the radius of curvature of the trajectory, and $K$ is a constant velocity gain factor. In our experiment, the $\alpha$ parameter was set to 0 as the trajectories had no inflection points. The $\beta$ exponent of the law, which is close to $\frac{1}{3}$ for adults, describes the observed regularity in upper limb movements. In the variable speed condition, the tangential velocity of stimulus movement was controlled by the two-thirds power law, thereby replicating biomechanically relevant movements dependent on the radius of curvature of the trajectory. This relationship was implemented for the movement of the elliptical tactile stimulus, where tangential velocity varied based on the radius of curvature along the trajectory. Conversely, in the constant speed condition, the stimulus moved at a fixed speed of approximately 6 cm/s.

The tactile pattern discrimination task was conducted using four different stimuli, derived from two shape and speed conditions. The two congruent stimuli were designed according to the two-thirds power law in terms of space-time: one presented a circular shape with a constant tangential displacement velocity (due to the constant radius of curvature), while the other exhibited an elliptical shape with a tangential velocity varying according to the same law. In contrast, the two incongruent stimuli were created by reversing the temporal characteristics of the congruent stimuli. In other words, the incongruent stimulus with a circular trajectory now had the cursor’s displacement velocity corresponding to that of the congruent elliptical stimulus, which varied. Conversely, the incongruent stimulus with an elliptical trajectory now had the cursor’s displacement velocity corresponding to that of the congruent circular stimulus, which remained constant.

Each stimulus consisted of 16 tactile points, evenly distributed along the trajectory, each with a 3 mm diameter, sequentially activated in a counterclockwise direction by the Ultrahaptics matrix, producing tactile stimuli of a discrete nature. Each tactile point remained active for approximately 200 ms(depending on the shape’s characteristics and the two-thirds power law). The activations of the tactile points were carefully coordinated to create continuous and smooth motion tailored to the two speed conditions. In the constant speed condition, stimulation times between each tactile point were adjusted to maintain a constant stimulus movement speed. Conversely, in the variable speed condition, stimulation times between tactile points were modulated in accordance with the two-thirds power law, replicating biomechanical movements dependent on the radius of curvature of the trajectory. Stimulation for each shape lasted approximately 2 s, allowing the stimulus to traverse the entire trajectory. To ensure consistent results and assess the reproducibility of participant responses, each stimulus was characterized by the presentation of two periods of tactile stimulation. This approach ensured a realistic and reliable tactile experience for each participant.

Before the start of the experiment, the area of the palm where stimulation was applied was individually specified for each participant and centered on the palm surface.

### Procedure

Participants took part in an approximately one hour and forty-five-minute laboratory experiment. Prior to the start of the experiment, each participant was seated at a distance of 40 cm from the screen with their right arm (up to the wrist) and fingertips immobilized on an armrest. This position was adopted to allow participants to keep their right hand steady, thus avoiding movement during the experiment and reducing muscle and joint contractions needed to maintain the position. The palm of the hand was positioned 20 cm above the UltraHaptics device (see Fig. 4). Participants were instructed to keep their right hand as still as possible throughout the experiment.

To begin, participants were familiarized with the two congruent stimuli, followed by a phase of thirty familiarization trials, presenting only the two congruent stimuli. This step aimed to acquaint participants with the tactile stimuli used in the experiment and ensure they understood the task correctly.

**Figure 5:**
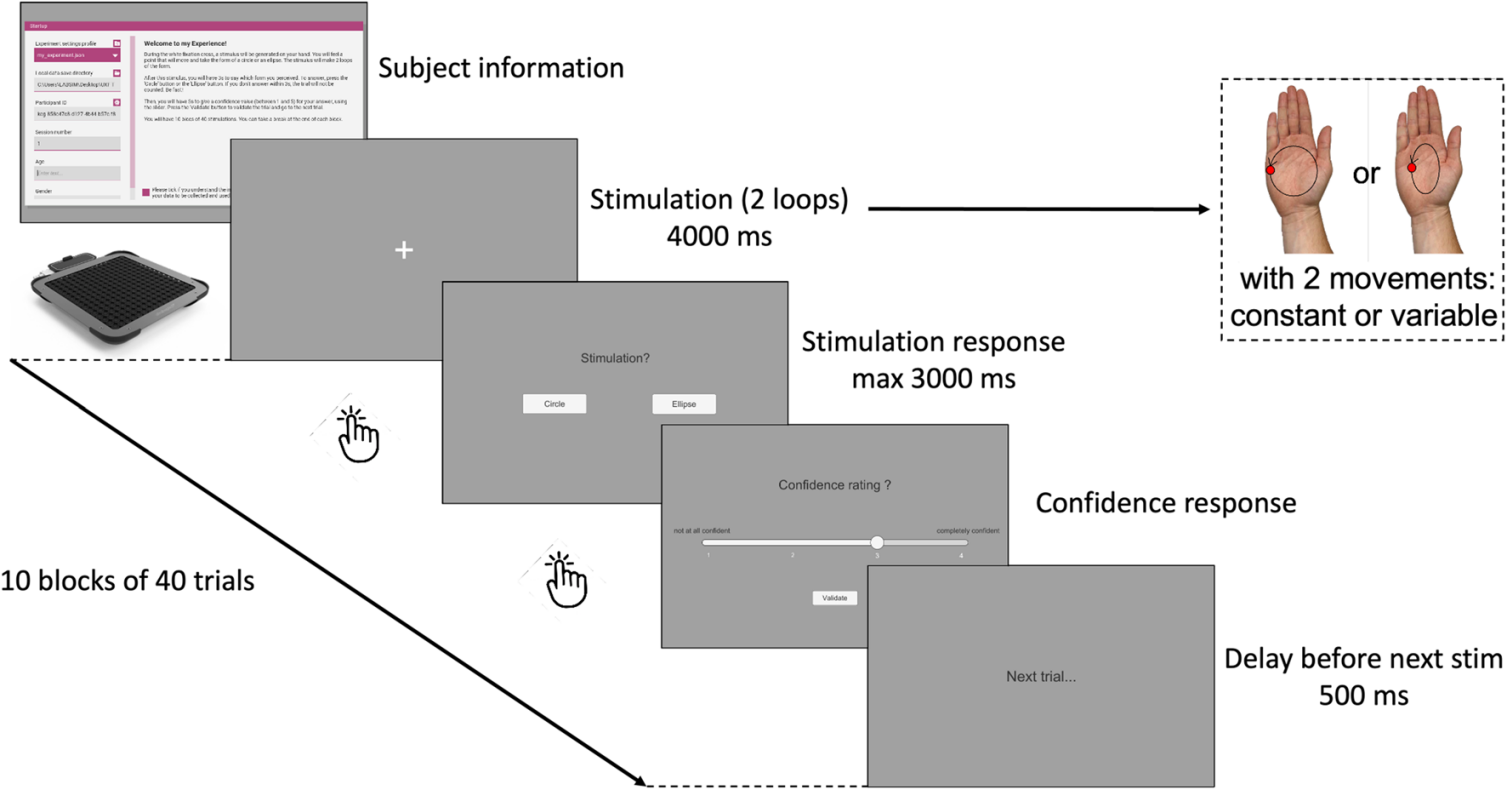
Overview of the experimental process with tactile air stimuli.

Next, participants completed the experimental phase, which included 400 trials randomizing congruent and incongruent stimuli, distributed across 10 blocks of 40 stimuli each (Fig. 5). Similar to the familiarization phase, participants had to identify whether the tactile stimulation presented corresponded to a circular or elliptical trajectory. Responses were provided using the touchscreen by pressing one of two buttons on the screen. Participants were required to make their response as soon as they had made their choice, and in all cases, within a time window of maximum 3 s. After each trial, upon providing their response, participants were required to estimate their confidence level on a scale of 1–4 in their response using a slider displayed on the screen and confirm it within 10 s. Unlike the familiarization phase, no feedback on tactile pattern discrimination performance was provided to participants during the experimental phase, and the interval between each trial was set at 0.5 s. Before each experimental block, the palm skin temperature of participants’ right hands was measured using the infrared thermometer. Finally, participants had the option to take short breaks between the blocks.

### Technical issue

During our study, we encountered a technical issue regarding the intensity level of stimuli generated by the UltraHaptics emitter. We noticed that the device’s Software Development Kit (SDK) was not specifically designed for experiments requiring a wide range of stimulus intensities. To ensure the most consistent stimulus intensity across all subjects, we decided to set the emitter’s intensity to maximum throughout the experiment. We indeed used the device with the amplitude modulation mode of control point within a range from 0 to 1, where 1 represents the maximum value of intensity that the device can produce. We adjusted this value to 1 because the perceived intensity below this value by the participants was quite low. In other words, when the intensity is set to 1, the acoustic pressure reaches approximately 155 dB, and the value of acoustic pressure is approximately 1120 Pa. The acoustic intensity associated with this acoustic pressure was approximately 3160 W/m^2^. This measure was taken to minimize the potential impact of stimulus intensity variation on the results of our study. Despite this technical limitation, we ensured that other aspects of the experiment remained rigorously controlled.

### Data processing

#### Discrimination performance

Mean discrimination performance (percentage of correct responses) was calculated across all trials in the experimental phase. Prior to conducting statistical tests, a Shapiro-Wilk test was performed, which indicated that the data did not follow a normal distribution. As a result, non-parametric tests were chosen for the analysis. Wilcoxon tests were conducted to compare performance between congruent and incongruent conditions. A Friedman test was also conducted to assess performance differences between different shape and speed conditions. Tests were performed with a significance threshold of p < 0.05. To evaluate significant differences between stimulus conditions, a multiple comparisons test (Bonferroni-corrected Wilcoxon test) was applied utilizing Kendall’s W effect size^43^ within the analysis. These analyses were conducted using the rstatix package^44^.

It is important to clarify that these analyses aimed to demonstrate that participants’ responses were not exclusively determined by the shape or speed of the stimuli, but rather by the congruent or incongruent nature between shape and speed of the stimulation. This clarification underscores the complexity of tactile perception and the nuanced ways in which congruency influences discrimination performance.

To determine if variations in hand skin temperature may have influenced discrimination performance, a Pearson correlation was conducted between temperatures measured before each block and participants’ discrimination performance.

#### Confidence—metacognitive efficiency

To assess participants’ subjective experience, a measure of confidence level in each response was recorded at the end of each trial. These measures reflect the participant’s confidence in their ability to distinguish stimuli. Wilcoxon tests were conducted on confidence levels in each congruence condition. However, because of known biases^22^, raw confidence measures cannot quantify the participant’s metacognitive accuracy, i.e., how accurately the participant judges their own performance. Therefore, confidence ratings collected after each perceptual decision were also analyzed to measure the efficiency of the metacognitive system. Metacognitive efficiency is assessed using the $M_{ratio}$^22,32^, which quantifies an observer’s ability to discriminate between correct responses and errors relative to their knowledge about the stimulus. In other words, it measures how aware a person is of their own cognitive abilities. The $M_{ratio}$ was calculated for each participant and in each condition separately using the metaSDT package^45^ in the R environment. A Bayes Factor on the paired t-test was also calculated using the BayesFactor package to confirm the absence of differences between conditions.

#### HDDM modeling of response time

**Figure 6:**
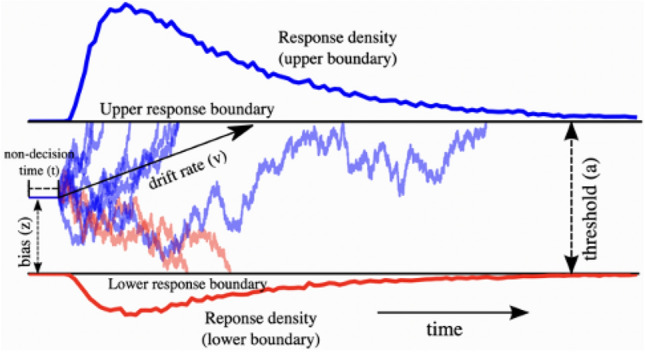
Trajectories of multiple drift processes^33^ (blue and red lines, middle panel). Evidence is accumulated over time (x-axis) with a drift rate v until one of the two boundaries (separated by threshold a) is crossed, triggering a response. The upper (blue) and lower (red) panels contain histograms of boundary crossing times for two possible responses.

Response time is an important indicator of performance in perceptual tasks. To understand the underlying mechanisms of perceptual decisions in our participants, we conducted Hierarchical Drift Diffusion Modeling (HDDM) analysis on the response times collected during the tactile discrimination task. The drift diffusion model is an approach used to model response times in decision tasks. It assumes that participants accumulate information over time until they reach a decision criterion, which determines their choice. This model allows the extraction of important parameters (Fig. 6), such as drift rate (the speed of evidence accumulation towards one or the other boundary, or the quality of accumulated evidence), decision threshold (the distance between the two boundaries or the amount of accumulated evidence), bias (the starting point of evidence accumulation, which is fixed and identical for all participants) and non-decision time (initial processing time). We fitted the Hierarchical Drift Diffusion Model (HDDM) to individual participant data using the HDDM package^33^ in the Python environment.

HDDM model analyses were conducted at various levels. We statistically tested the drift rates and thresholds of congruence and incongruence using a within-subject model with a paired t-test. And to deepen our understanding, a repeated measures ANOVA was conducted to evaluate the evidence accumulation differences for our stimuli for each participant. To assess significant differences between each of the stimuli, we used pairwise comparison tests employing the Bonferroni correction method for multiple test adjustments. The results of this analysis help us better understand the underlying cognitive processes involved in tactile discrimination and identify what determines significant differences that may exist between congruent and incongruent conditions, as well as our stimuli.

### Ethics declarations

All participants provided informed consent to participate in the study and to publish their images.

## Data Availability

The datasets generated during and/or analyzed during the current study are available from the corresponding author on reasonable request.
